# The Construction and Immunogenicity Analyses of Recombinant Pseudorabies Virus With NADC30-Like Porcine Reproductive and Respiratory Syndrome Virus-Like Particles Co-expression

**DOI:** 10.3389/fmicb.2022.846079

**Published:** 2022-03-02

**Authors:** Jun Zhao, Ling Zhu, Lei Xu, Fengqing Li, Huidan Deng, Yao Huang, Sirui Gu, Xianggang Sun, Yuancheng Zhou, Zhiwen Xu

**Affiliations:** ^1^College of Veterinary Medicine, Sichuan Agricultural University, Chengdu, China; ^2^Key Laboratory of Animal Disease and Human Health of Sichuan Province, Chengdu, China; ^3^Animal Breeding and Genetics Key Laboratory of Sichuan Province, Sichuan Animal Science Academy, Chengdu, China

**Keywords:** PRV variant, NADC30-like PRRSV, VLPs, viral-vectored vaccine, immunogenicity analyses

## Abstract

Porcine reproductive and respiratory syndrome (PRRS) and pseudorabies (PR) are highly infectious swine diseases and cause significant financial loss in China. The respiratory system and reproductive system are the main target systems. Previous studies showed that the existing PR virus (PRV) and PRRS virus (PRRSV) commercial vaccines could not provide complete protection against PRV variant strains and NADC30-like PRRSV strains in China. In this study, the PRV variant strain XJ and NADC30-like PRRSV strain CHSCDJY-2019 are used as the parent for constructing a recombinant pseudorabies virus (rPRV)-NC56 with gE/gI/TK gene deletion and co-expressing NADC30-like PRRSV GP5 and M protein. The rPRV-NC56 proliferated stably in BHK-21 cells, and it could stably express GP5 and M protein. Due to the introduction of the self-cleaving 2A peptide, GP5 and M protein were able to express independently and form virus-like particles (VLPs) of PRRSV in rPRV-NC56-infected BHK-21 cells. The rPRV-NC56 is safe for use in mice; it can colonize and express the target protein in mouse lungs for a long time. Vaccination with rPRV-NC56 induces PRV and NADC30-like PRRSV specific humoral and cellular immune responses in mice, and protects 100% of mice from virulent PRV XJ strain. Furthermore, the virus-neutralizing antibody (VNA) elicited by rPRV-NC56 showed significantly lower titer against SCNJ-2016 (HP-PRRSV) than that against CHSCDJY-2019 (NADC30-like PRRSV). Thus, rPRV-NC56 appears to be a promising candidate vaccine against NADC30-like PRRSV and PRV for the control and eradication of the variant PRV and NADC30-like PRRSV.

## Introduction

Porcine reproductive and respiratory syndrome (PRRS) appeared in Europe and the United States in the 1990s and became a problem that plagued the global pig industry ever since. PRRS virus (PRRSV) is a single-stranded positive-strand RNA virus with capsule, belonging to the arteritis virus family and arteritis virus genus, with a diameter of 50–65 nm. The pig is the natural host of PRRSV. The lungs are the main target organ *in vivo*, and porcine alveolar macrophages (PAMs) are the main target cells of PRRSV infection *in vitro*. Heparin sulfate, vimentin, CD151, CD169, and CD163 are the main receptors of PRRSV infection (Nan et al., 2017). Because of the great differences in genes and proteins between the PRRSV-1 and PRRSV-2, they are divided into two species (Kuhn et al., 2016). According to the classification system, the PRRSV-1 was divided into three subtypes (subtypes 1–3), and the PRRSV-2 was classified into nine lineages with several sublineages in each lineage (Shi et al., 2010a,b). The main prevalent PRRSV-2 strains in China are types 1, 3, 5, 8, and 9 (Chen et al., 2019; Han et al., 2020). Lineage 1 PRRSV contains representative strains, such as NADC30, JL580, NADC34, and RFLP 1-4-4. Lineage 3 PRRSV is mainly prevalent in South China and has low pathogenicity, including representative strains such as QYYZ and GM2. Lineage 5 mainly contains the classical PRRSV strain represented by VR-2332. Lineage 8 contains highly virulent strains represented by TJ, JXA1, TA-12, and classical PRRSV strains represented by CH-1a. Lineage 9 was discovered in Xinjiang in 2011. Since the emergence of PRRSV strains in China, the most popular strains are lineage 8, including classic strain (CH-1a) and HP-PRRSV in 2006 (Deng et al., 2012). The NADC30-like strain in lineage 1 appeared in the United States in 2008 and in China in 2013. Since then, the detection rate of clinical samples in nine provinces such as North China, East China, South China, and Central China has gradually increased (Guo et al., 2018, 2019; Jiang et al., 2020). The results of challenge protection experiment using NADC30-like PRRSV strains showed that the most widely used commercial vaccines in China cannot effectively protect pigs from the attack of NADC30-like PRRSV strain. Pigs infected with NADC30-like PRRSV strain after vaccination still have obvious clinical symptoms, high viremia, and virus load in various tissues (Bai et al., 2016; Zhou et al., 2017; Wei et al., 2019; Chen et al., 2021). At present, there is no commercial PRRSV vaccine developed with NADC30-like PRRSV strain. Therefore, the development of a PRRSV vaccine against NADC30-like PRRSV is particularly important to combat the continuously increasing epidemic of NADC30-like PRRS outbreaks.

Virus-neutralizing antibodies, the key weapon of the body to resist virus infection, is the main evaluation index of many effective vaccines to prevent virus infection (Robinson et al., 2015). The PRRSV virion surface contains at least seven envelope proteins, GP2a, GP2b, GP3, GP4, GP5, M, and N protein, respectively. GP5 and M protein are the major envelope proteins, and they are disulfide-linked heterodimers. The minor glycoprotein GP2a, GP3, and GP4 form noncovalent heterotrimers (Trible et al., 2015). Multiple previous studies have identified multiple neutralization epitopes distributed on major structural protein (GP5 and M) and minor glycoprotein (GP2a, GP3, and GP4) (Vanhee et al., 2011). In the last years, a lot of effort has been put into VLPs for vaccine development to control human or animal disease (Kang et al., 2021; Lampinen et al., 2021; Marin et al., 2021; Wang et al., 2021). Previous studies showed that GP5 and M protein interaction, GP2, GP3, GP4, and E protein interaction, GP5, GP4, GP3, GP2a, and M protein interaction, and GP5, M, and N protein interaction of PRRSV could form VLPs (Uribe-Campero et al., 2015; Binjawadagi et al., 2016; García Durán et al., 2016).

Pseudorabies virus (PRV) belongs to the family Herpesviridae, subfamily Alphaherpesvirinae (Müller et al., 2011; Tang Y. D. et al., 2016) and can infect pigs, cattle, sheep, and many other livestock and wild animals, causing fever, severe itching, and encephalomyelitis in infected animals. Pigs are the main reservoir host and infection source of PRV, and are threatened by pseudorabies (PR) at all ages (Müller et al., 2011; Zhang et al., 2015). PRV infection mainly causes disorder of the nervous system and respiratory system in piglets and the abortion of pregnant sows (Weigel et al., 2003; Wang et al., 2015; Lamote et al., 2016; Sun et al., 2016). In the 1970s, The Bartha-K61 vaccine was imported into China from Hungary and effectively controlled the prevalence of PR (Tong et al., 2015). However, variant PRV emerges in many Bartha-K61 vaccinated farms in the Northern provinces of China since 2011 (An et al., 2013; Gu et al., 2018).

Pseudorabies virus is a double-stranded DNA virus of approximately 150 kb in length, which contains almost 70 open reading frames (ORFs) that encode 70–100 viral proteins including structural, nonstructural, and virulence-associated protein (Klupp et al., 2004). The large genome of the PRV contains many nonessential genes with many insertion sites that can integrate and express heterologous genes, including the TK, gE, gI, and gG genes. The absence of these nonessential genes results in a diminished virulence phenotype of the virus while having no significant effect on the immune response (Klupp et al., 2004; Olsen et al., 2006). At present, PRV has developed into a powerful vector system for expressing exogenous protein, which can carry and express the main immunogenic genes and protein of other viruses, such as PCV2 Cap, PCV3 Cap, PPV VP2, ASFV CD2v, HP-PRRSV GP5, and M proteins (Freuling et al., 2017; Feng et al., 2020; Zheng et al., 2020; Yao et al., 2021). These bivalent or trivalent recombinant vaccines developed with a PRV live virus vector can stimulate the body to produce a specific immune response to the target virus without affecting the autogenous immune effect of PRV. In this study, based on the functional characteristics of PRRSV GP5 and M proteins, we construct a recombinant PRV strain, named recombinant pseudorabies virus (rPRV)-NC56. It expresses the NADC30-like PRRSV GP5 and M proteins stably, and the two proteins assemble into VLPs intracellularly. The safety and ability to elicit humoral and cellular immune responses of rPRV-NC56 were evaluated in mice to provide evidence for the future vaccine development against both NADC30-like PRRSV and variant PRV.

## Materials and Methods

### Viruses, Cells, and Plasmids

PRV-XJ (Genbank accession No. MW893682.1) strain was obtained from specimens of pseudorabies-infected pigs at Sichuan Province in 2015 by our lab, and it has been proven to be a potential PR vaccine with good immunogenicity (Yin et al., 2017). PRV XJ-ΔgE/gI/TK-EGFP was built and stored in our lab. All viruses were propagated in BHK-21 cells in Dulbecco’s modified Eagle’s medium (DMEM) (12100046, Gibco) supplemented with 10% fetal bovine serum (10099133C, Gibco). Baby hamster Syrian kidney cells (BHK-21) and HEK293T cells were purchased and cultured in DMEM medium containing 10% fetal bovine serum in our lab. The single-guide RNA (sgRNA) lentiCRISPR V2 plasmid of gE gene (GGGCAGGAACGTCCAGATCC), TK gene (CTCGACGGCGCCTACGGCAC, GCCGCGTAC GGCGACCACATC), and pEGFP-gI/28K plasmid containing gI and 28K homologous arms of PRV were constructed by our lab.

The NADC30-like PRRSV ORF5 and ORF6 genes were amplified from the CHSCDJY-2019 strain genome (Genbank accession No. MT075480.1) (Zhao et al., 2021). The nucleotide sequences of self-cleaving T2A and P2A peptides were added to the donor template by PCR amplification, and the donor template (T2A-ORF6-P2A-ORF5) was obtained by fusion PCR amplification. The recombinant transfer plasmid (pEGFP-2A-NC-OFR5-6) was constructed by Seamless Cloning Kit (D7010FT, Beyotime) with the donor template and the pEGFP-gI/28K (digestion with EcoR I and Bcl I; 1611 and 1045A, Takara), and named as pEGFP-2A-NADC30-like-ORF6-ORF5 (pEGFP-2A-NC-OFR5-6) (Supplementary Figure 1). All the sequences of the primers are listed in Supplementary Table 1.

### Generation of Recombinant Virus and PCR Identification

The PRV-XJ genomic DNA was extracted according to the Omega DNA extraction kit instructions. PRV-XJ genomic DNA (3 μg), pEGFP-2A-NC-OFR5-6 plasmid (5 μg), and sgRNA-gE plasmid (5 μg), with a molar ratio of 20:1, were cotransfected into the well-developed HEK293T cells using Lipofectamine 3000 Transfection Reagent (L3000015, Thermo). After transfection, the cells were cultured under 37°C until cytopathic effect (CPE) occurred. Virus supernatant was taken to inoculate BHK-21 cells, green fluorescence plaques were picked out, and three rounds of plaque purification were performed. The purified viruses were named rPRV-ΔgE/gI-NC-ORF5-6. The rPRV-ΔgE/gI-NC-ORF5-6 viral DNA was extracted. The rPRV-ΔgE/gI-NC-ORF5-6 (3 μg) and two sgRNA of TK genes (5 μg each), with a molar ratio of 20:1, were cotransfected into the well-developed HEK293T cells using Lipofectamine 3000 transfection Reagent. Pure recombinant PRV were plaque purified, named as rPRV-NC56.

The viral DNA of the purified virus rPRV-NC56 and PRV-XJ was extracted by the Genomic DNA Purification Kit (K182001, Thermo). The TK, gE, and gB genes and insert nucleic acid sequence (ORF6-P2A-ORF5) were detected by PCR. The detection primers are shown in Supplementary Table 1. The PCR products were agarose gel electrophoresis, the target bands were recovered and purified, and sequencing was done by Sangon Biotech (Shanghai) Co., Ltd.

### Immunofluorescence Assay for GP5 and M Protein Expression Detection

The purified recombinant virus rPRV-NC56 was inoculated in a 12-well plate filled with monolayer BHK-21 cells with 0.05 MOI. Furthermore, an equal amount of parental involvement strain PRV XJ was set up as a negative control. The BHK-21 cells were fixed with paraformaldehyde, permeabilized with 0.2% X-Triton (9002-93-1, Sangon Biotech), followed by incubation with rabbit anti-GP5 polyclonal (bs-4504R, Bioss) or rabbit anti-M polyclonal antibody (bs-4507R, Bioss) for 1 h at 37°C, respectively. After three washes with PBS (20012050, Gibco), the cells were stained with corallite 594-conjugated goat anti-rabbit IgG (SA00013-4, Proteintech) and DAPI (C0060, Solarbio) (0.5 μg/ml) for 30 min at 37°C, and cells were washed with PBS three times. The GP5 and M proteins were observed under an inverted fluorescence microscope.

### Western Blotting Analysis for GP5, M, gE, and gB Protein Expression Detection

The rPRV-NC56 and PRV XJ were, respectively, inoculated in a six-well plate for the fifth generation with 0.1 MOI. After 36 h, the BHK-21 cells were lysed with RIPA lysis solution (P0013B, Beyotime), and the supernatant was collected by centrifugation at 12,000 r/min for 5 min. Then the supernatant was boiled with 5× SDS-PAGE loading buffer for 20 min and centrifuged at 15,000 r/min for 15 min. After 12% SDS-PAGE electrophoresis (1610185, Bio-Rad Laboratories), the gel was electrotransferred to a nitrocellulose membrane (1620117, Bio-Rad Laboratories). Membranes were blocked with 5% skim milk in PBS for 1 h at 37°C. After three washings with PBST (PBS containing 1% Tween 20), the nitrocellulose membranes were, respectively, incubated overnight at 4°C with a primary antibody. The primary antibodies used in this study were rabbit anti-PRRSV GP5 polyclonal antibody (bs-4504R, Bioss), rabbit anti-RRRSV M polyclonal antibody (bs-4507R, Bioss), mouse anti-PRV gE monoclonal antibody (82289, TianTech), anti-PRV gB (The gB polyclonal antibodies were obtained from rabbits immunized with gB protein in our lab), and rabbit anti-β-actin monoclonal antibody (66009-1-Ig, Proteintech). After three washings with PBST, nitrocellulose membranes were incubated with horseradish peroxidase-conjugated goat anti-mouse or goat anti-rabbit secondary antibody (D110087 and D110058, Sangon Biotech) for 1 h at room temperature, respectively, then washed with PBST, and developed using SuperSignal™ West Pico PLUS chemiluminescent substrate (34580, Thermo) according to the manufacturer’s instructions.

### Virus One-Step Growth Curve and Plaque Assays

The virus growth kinetics of rPRV-NC56, rPRV XJ-ΔgE/gI/TK-EGFP, and PRV-XJ was compared. The 0.1 MOI virus was inoculated when the growth density reached 80% of BHK-21 cells in T25 cell culture flask. The cell supernatants were collected at different time points (0, 4, 8, 12, 24, 36, 48, 64 hpi) after inoculation; the TCID_50_ of the viruses was measured. Three replicated tests were taken for each sample, and the values were averaged to draw a one-step growth curve.

The 100 TCID_50_ of rPRV-NC56, rPRV XJ-ΔgE/gI/TK-EGFP, and parental virus PRV-XJ were placed into six-well plates full of BHK-21 cells, respectively. After incubating at 37°C for 1 h, the cells were covered with DMEM containing 1% low-melting point agarose. The infected BHK-21 cells were maintained at 37°C and 5% CO_2_ for 96 h, and were stained with formaldehyde–crystal violet staining solution containing 1% crystal violet and 4% formaldehyde. The morphology and size of virus plaques were analyzed using Image Pro Plus (v 7.0).

### Transmission Electron Microscopic Observation of Porcine Reproductive and Respiratory Syndrome Virus-Like Particles

A 1/1,000 dose of the rPRV-NC56, rPRV XJ-ΔgE/gI/TK-EGFP, PRV-XJ, and CHSCDJY-2019 was, respectively, inserted into a T75 cell culture flask full of monolayer Marc-145 cells, and the cells were collected in a centrifuge tube when they were more than 80% diseased. Cells were centrifuged at 1,500 r/min for 10 min, and the supernatant was discarded. Glutaraldehyde fixative (0.5%) was slowly added along the tube wall and was left for 10 min at 4°C. Then it was centrifuged at 15,000 r/min for 15 min, the supernatant was discarded, and 3% glutaraldehyde fixative was slowly added to fix it. The cell culture clumps after fixation were sent to Chengdu Lilai Biological Co., Ltd. The formation of rPRV-NC56, rPRV XJ-ΔgE/gI/TK-EGFP, PRV-XJ, and CHSCDJY-2019 strains and the formation of PRRSV VLPs were observed under an electron microscope. To assess the formation and release of VLPs, infections with rPRV-NC56 and CHSCDJY-2019 were performed in Marc-145 cells. The VLPs were condensed from the supernatant by 100-KDa MWCO centrifugal concentrator (UFC9100, Millipore), and the unassembled GP5 and M proteins were removed. All the fractions obtained were analyzed by SDS-PAGE and Western blot.

### Safety, Immunogenicity, and Challenge in Mice

Ninety healthy BALB/c mice (25 g) were randomly divided into nine groups (10 mice/each). Groups A–C, D–F, G–H, and I were inoculated with different concentrations of rPRV-NC56 (10^6.0^, 10^7.0^, and 10^8.0^ TCID_50_), rPRV XJ-ΔgE/gI/TK-EGFP (10^6.0^, 10^7.0^, and 10^8.0^ TCID_50_), PRV-XJ (10^2.0^ and 10^3.0^ TCID_50_), and DMEM with intramuscularly (i.m.) (200 μl/dose), and were continuously observed for 14 days. Blood was collected from the tail vein of 10 mice in each group at 1 and 3 days for IL-6 and TNF-α (KMC0061 and BMS607-3, Thermo) detection, respectively.

Sixty BALB/c mice were divided into two groups: 30 mice in one group were immunized with rPRV-NC56 [i.m. at 200 μl/dose (10^6^ TCID_50_)], and 30 mice in the other groups were injected with equal volume of PBS. The immunized mice were killed at 1-day postvaccination (dpv), 3 dpv, 5 dpv, 7 dpv, and 21 dpv, and the lung tissues were collected for PRRSV GP5 protein immunohistochemistry (IHC) detection and PRV gB gene real-time quantitative polymerase chain reaction (qPCR) detection. The PRV gB segment was cloned into the PMD-18T vector (6011, TAKARA) to construct the PMD18T-gB recombinant plasmid and generate a standard curve. gB forward, 5′-CGGCAAGTGCGTCTCC-3′ and reverse, 5′-TGTAGGTGTCGTTGGTGGTG-3′. The IHC detection was sent to Chengdu Lilai Biological Co., Ltd., and 8–12 fields under ×400 magnification were selected for each sample to count the positive cells using image Pro Plus (V 7.0).

Seventy-two BALB/c mice were randomly divided into three groups of 24 each, and the three groups were inoculated with rPRV-NC56 and rPRV XJ-ΔgE/gI/TK-EGFP and DMEM, respectively. The viruses were intramuscularly injected into mice at a dose of 200 μl (10^6^ TCID_50_), and the other one group was injected with 200 μl of DMEM as control. Three groups were booster immunized with the same dose 2 weeks postvaccination (wpv). Blood was collected from the tail vein of 10 mice in each group at 1–6 weeks after the first immunization. Levels of PRV gB, NADC30-like PRRSV GP5, and M-specific antibodies in the serum were measured by enzyme-linked immunosorbent assay (ELISA). The splenocytes from 10 mice at 4 wpv were isolated and, respectively, stimulated *in vitro* with 10^6^ TCID_50_ of UV-inactivated CHSCDJY-2019 (NADC30-like PRRSV) and SCNJ-2016 (HP-PRRSV) strains as the antigen. Meanwhile, the specific CD8 T lymphocytes were detected by cell proliferation assay. The secretion of IL-2, IL-4, and IFN-γ (BMS601, BMS613, and BMS606-2, Thermo) in the blood of mice on weeks 2, 4, and 6 were measured by cytokine assay kits.

The mice in each group were challenged at week 8 after primary vaccination via footpad injection of 10× LD_50_ of the PRV XJ (LD_50_ = 10^2.74^ TCID_50_/ml). The serum of each group of mice was collected at 3 days postchallenge (dpc) to detect IL-6 and TNF-α (KMC0061 and BMS607-3, Thermo). All mice were monitored for 14 days after the challenge and humanely euthanized. The main organs and tissues of each group of mice were sent to Chengdu Lilai Biological Co., Ltd. for hematoxylin–eosin (HE) staining and histopathological observation after fixing with 4% paraformaldehyde.

### Enzyme-Linked Immunosorbent Assay for Antibodies and Cytokine

The serum of each group of mice was collected and subjected to anti-gB antibody by commercial ELISA kit (IDEXX, United States). Mouse IL-2, IL-4, IFN-γ, TNF-α, and IL-6 ELISA kits were purchased from Thermo Fisher Scientific (MA, United States). All operations were carried out according to the instructions of commercial ELISA kit. NADC30-like PRRSV anti-GP5 and anti-M antibody were detected by NADC30-like GP5 and M antibody ELISA assays constructed in our lab (Zhang, 2021).

### Neutralizing Antibody Assay

BHK-21 or Marc-145 cells were cultured in 96-well plates. The serum was inactivated at 56°C for 30 min, and serial double dilutions (1:2n) were mixed with an equal volume of the PRV-XJ or PRRSV CHSCDJY-2019 and SCNJ-2016 containing 200 TCID_50_; the mixture was incubated at 37°C for 1 h. The mixture was incubated with BHK-21 or Marc-145 cells in a 96-well plate at 37°C for 5–7 days. The CPE in each pore was observed and counted under inverted microscope. Neutralizing antibody titers were calculated as the average of three measurements according to the Reed–Muench method (Hong et al., 2007; Robinson et al., 2015).

### Flow Cytometry

In order to detect the number of CD3^+^/CD4^+^/CD8^+^ cells in the spleen of mice, the spleen cells of mice were isolated at 2 wpv after primary immunization. The dispersed spleen cells were adjusted to 10^7^/ml and mixed with the PE-Cy anti-mouse CD8α (70-AM008A04-100, MULTI), FITC anti-mouse CD4 (70-AM00401-100, MULTI), and APC Hamster Anti-Mouse CD3ε (70-AM003E07-100, MULTI) for 30 min at 37°C. Subsequently, the cells were washed three times with PBS and analyzed by flow cytometry.

### Statistical Analysis

All data are expressed as the means ± standard errors (SEs). The data analysis between different groups was analyzed by two-tailed Student’s *t*-test and/or one-way analysis of variance (ANOVA) of the GraphPad Prism 8.0 software. A value of *p* ≤ 0.05 was considered statistically significant (*p < 0.05, ^**^p < 0.01, and ^***^p < 0.001).

## Results

### Generation and Characterization of the Recombinant Pseudorabies Virus-NC56

**FIGURE 1:**
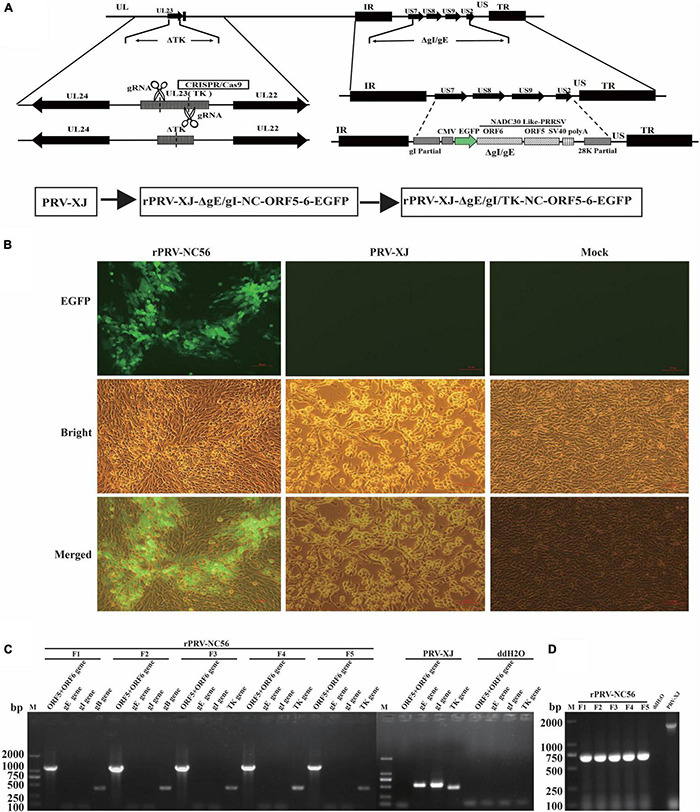
Construction of recombinant pseudorabies virus (rPRV)-NC56. **(A)** Schematic diagram of recombinant pseudorabies virus (PRV) construction strategy. **(B)** Baby hamster Syrian kidney (BHK-21) cells infected with rPRV-NC56 and PRV XJ were observed under fluorescence microscope. **(C)** PCR identification results of the gE, gI, gB, TK, and partial inserted target genes (ORF5-6) of rPRV-ΔgE/gI-NC-ORF5-6 cultures. **(D)** PCR identification of the CRISPR/Cas9 deletion of the TK gene.

The target gene (CMV-EGFP-T2A-ORF6-P2A-ORF5-SV40) was inserted in the location of gE and gI gene deletion of PRV XJ strain by CRISPR/Cas9-mediated homologous recombination, and the TK gene was deleted by CRISPR/Cas9 (Figure 1A). The inserted target fragment is driven by CMV endogenous promoter, and the independent expression of EGFP, M, and GP5 proteins is realized by ribosomal jump translation of 2A cleavage peptide. The first generation of CRISPR/Cas9-mediated recombinant viruses (rPRV-ΔgE/gI-NC-ORF5-6) were harvested after infection with a 0.1 MOI of PRV XJ, which showed the highest efficiency for gene recombination. rPRV-ΔgE/gI-NC-ORF5-6 proliferated continuously in BHK-21 cells for five generations. PCR detection results of the five generations of rPRV-ΔgE/gI-NC-ORF5-6 cultures showed that gE and gI genes were negative, and gB, TK, and partial inserted target genes (ORF6-P2A-ORF5) were positive (Figure 1C). The TK gene of rPRV-ΔgE/gI-NC-ORF5-6 was deleted by CRISPR/Cas9, and the recombinant virus rPRV-ΔgE/gI/TK-NC-ORF5-6 (rPRV-NC56) was obtained (Figure 1B). The results of PCR detection of TK gene of F1–F5 generations of rPRV-NC56 and PRV-XJ showed that there were 661- and 1,579-bp charged swimming bands, respectively (Figure 1D).

**FIGURE 2:**
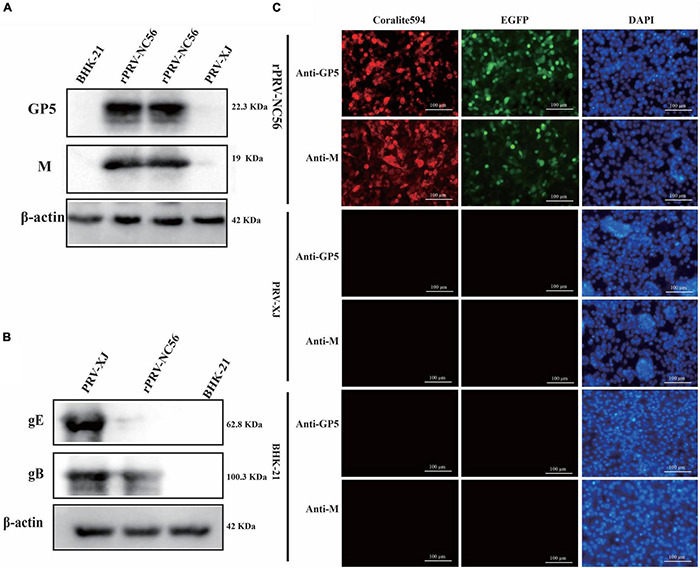
Western blot and immunofluorescence assay (IFA) identification of recombinant pseudorabies virus rPRV-NC56. **(A)** Western blotting analysis of NADC30-like porcine reproductive and respiratory syndrome virus (PRRSV) GP5 and M proteins in BHK-21 cells infected with rPRV-NC56. **(B)** Western blotting analysis of PRV gE and gB protein in BHK-21 cells infected with rPRV-NC56. **(C)** Detection of the GP5 and M protein expression through an IFA. Green, EGFP; red, GP5 protein-positive cells; blue, DAPI-stained BHK-21 cell nucleus. DAPI, 4′,6-diamidino-2-phenylindole.

The NADC30-like PRRSV GP5, M protein expression in BHK-21 cells that were infected with rPRV-NC56 were confirmed by indirect immunofluorescence assay (IFA) and Western blot. The expression of PRV gE and gB proteins of the recombinant virus was verified by Western blot. The PRV XJ strain was used as the control. For assessment of the NADC30-like PRRSV GP5 and M protein and PRV gE, gB protein expression, BHK-21 cells infected with rPRV-NC56 or PRV XJ were lysed by RIPA lysis solution, and the cell lysates were analyzed by Western blot. The expression of β-actin was detected in all groups with Western blot. The band specifically recognized by the rabbit anti-GP5 and anti-M antibody appeared in the BHK-21 cells infected with rPRV-NC56 but not in those infected with PRV XJ strain (Figure 2A). The band specifically recognized by the rabbit anti-gB antibody appeared in the BHK-21 cells infected with rPRV-NC56 and PRV XJ strain. The band specifically recognized by the rabbit anti-gE antibody showed in the BHK-21 cells infected with PRV XJ strain but not in those infected with rPRV-NC56 (Figure 2B). The expression of GP5 and M proteins in rPRV-NC56- and PRV XJ-infected BHK-21 cells was identified by IFA. The identification results showed that rPRV-NC56-infected BHK-21 cells were positive and PRV XJ-infected BHK-21 cells were negative (Figure 2C).

### Biological Characteristics of Recombinant Pseudorabies Virus-NC56

**FIGURE 3:**
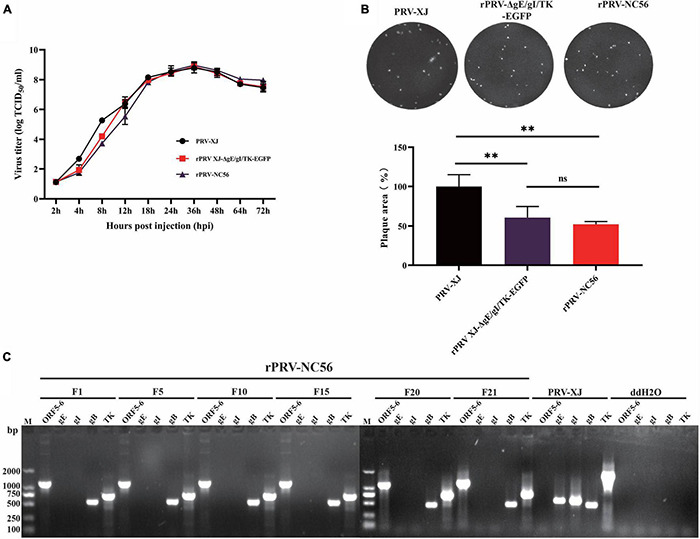
Biological characterization of recombinant pseudorabies virus rPRV-NC56. **(A)** One-step growth curves of PRV XJ, rPRV XJ-ΔgE/gI/TK-EGFP, and rPRV-NC56 in BHK-21 cells. **(B)** Comparison of plaque size of BHK-21 cells infected with PRV XJ, rPRV XJ-ΔgE/gI/TK-EGFP, and rPRV-NC56. **(C)** PCR identification of gE, gI, gB, TK, and a partially inserted target gene (ORF5-6) in cell cultures from generations 1, 5, 10, 15, 20, and 21 of rPRV-NC56. **p < 0.01.

We analyzed the one-step growth curves and plaque experiments of rPRV-NC56, rPRV XJ-ΔgE/gI/TK-EGFP, and PRV XJ strains to determine whether the insertion and expression of foreign fragments affect the biological characteristics of the virus. One-step growth curve showed that within 18 h after rPRV-NC56 infected BHK-21 cells, the virus titer in the supernatant was lower than that of the parent strain PRV-XJ and rPRV XJ-ΔgE/gI/TK-EGFP, and from 24 h after infection, the titer of rPRV-NC56 was similar to those of PRV-XJ and rPRV XJ-ΔgE/gI/TK-EGFP in BHK-21 cells (Figure 3A). The empty spot areas of rPRV-NC56 and rPRV XJ-ΔgE/gI/TK-EGFP were not significantly different, but they have a plaque size of about 60% of the parental PRV XJ strain (Figure 3B). In addition, the gene deletion and the stability of the foreign gene carrying the recombinant virus (F1–F21) were verified by PCR. The recombinant viruses were identified by PCR for deletion of gE, gI, and TK genes. There were no electrophoretic bands for gE, gI genes, and the TK gene and inserted foreign gene (ORF6-P2A-ORF5), respectively, showed an electrophoretic band of about 661 and 1,191 bp, which was consistent with the expected results, indicating that gE, gI, and TK genes were effectively absent, and the inserted foreign gene was stably carried (Figure 3C).

### Observation of Recombinant Pseudorabies Virus-NC56 Virus Particles and Porcine Reproductive and Respiratory Syndrome Virus-Like Particles by Transmission Electron Microscopic

**FIGURE 4:**
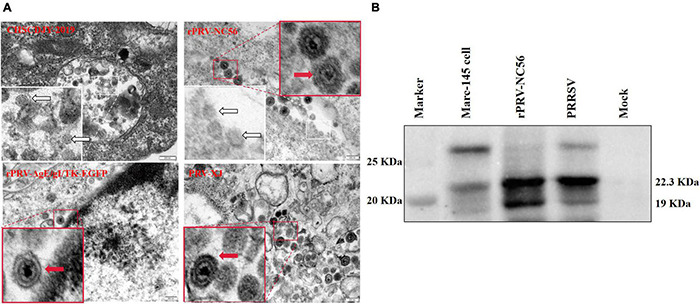
Transmission electron microscopy analysis of PRRSV, PRV XJ, rPRV XJ-ΔgE/gI/TK-EGFP, and rPRV-NC56 in Marc-145 cells. **(A)** PRRSV CHSCDJY-2019 PRV XJ, rPRV XJ-ΔgE/gI/TK-EGFP, and rPRV-NC56 in Marc-145 cells. The red arrow indicates PRV virus particles, and the white arrow indicates PRRSV virus particles and virus-like particles. **(B)** PRRSV GP5 and M proteins in ultrafiltration cell culture supernatant of Marc-145 cells infected with rPRV-NC56 were identified by Western blotting. Virus-like particles (VLPs) assembled by GP5 and M proteins will be intercepted by ultrafiltration column (100 kDa).

The difference of virus particle structure between rPRV-NC56, rPRV XJ-ΔgE/gI/TK-EGFP, and PRV XJ strains, and the formation of VLPs by NADC30-like PRRSV GP5 and M proteins expressed by the rPRV-NC56 virus was investigated by an electron microscope. As in Figure 4A, the mature envelope PRV virus particles with the size of about 120–130 nm were found in the cytoplasm in Marc-145 cells infected with rPRV-NC56, rPRV XJ-ΔgE/gI/TK-EGFP, and PRV XJ. A similar morphology of viral particles was found to exist in all three groups and show the typical structure of PRV. Mature PRRSV virus particles with a size of about 40–60 nm can be found in the vesicles of Marc-145 cells infected by PRRSV CHSCDJY-2019. Interestingly, mature PRV virus particles of about 120–130 nm and nucleic acid-free 40- to 60-nm PRRSV VLPs can concurrently be found in the vesicles of BHK-21 cells infected with rPRV-NC56 (Figure 4A). The specific bands of GP5 and M proteins were detected in the rPRV-NC56 infection group and the CHSCDJY-2019 infection group in the supernatant of Marc-145 cells infected with rPRV-NC56 and CHSCDJY-2019 (Figure 4B).

### Safety of Recombinant Pseudorabies Virus-NC56 in Mice

**FIGURE 5:**
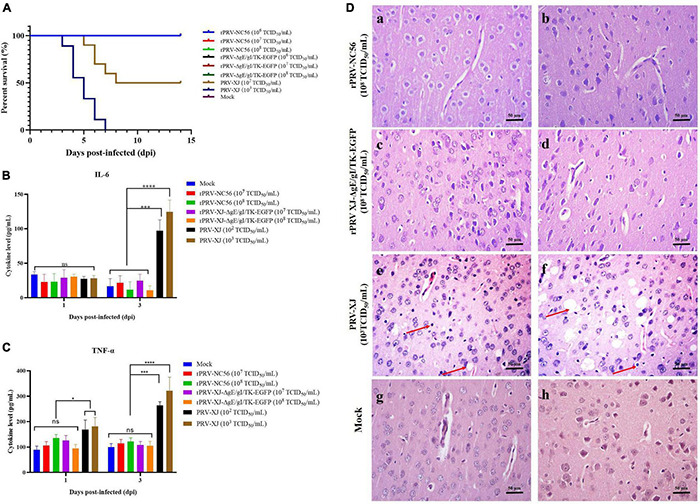
The safety assessment of rPRV-NC56. **(A)** Survival rate of mice infected with different titers of PRV XJ, rPRV XJ-ΔgE/gI/TK-EGFP, and rPRV-NC56. **(B)** Plasma IL-6 was detected by enzyme-linked immunosorbent assay (ELISA). **(C)** Plasma TNF-α was detected by ELISA. **(D)** Pathological observation of HE staining in brain tissue of mice infected with PRV XJ, rPRV XJ-ΔgE/gI/TK-EGFP, and rPRV-NC56 (×400). a, c, and e were granulocyte layers in the cerebral cortex, and b, d, and f were the pyramidal cell layers in the cerebral cortex. *p < 0.05; ^**^p < 0.01; ^***^p < 0.005; and ^****^p < 0.001. Neuronal necrosis and vacuolar degeneration are indicated by red arrows.

We used mice to evaluate the safety of the recombinant virus, and the results are shown in Figure 5.

After injection with different doses of rPRV-NC56, rPRV XJ-ΔgE/gI/TK-EGFP, and DMEM culture medium, the mice showed no clinical symptoms and death during the observation period (14 days). The mice injected with 10^3^ TCID_50_/ml of PRV XJ showed itching and bite symptoms, and all of them died within 7 days. The 10^2^ TCID_50_/ml of PRV XJ-infected mouse group died (50%) within 8 days (Figure 5A). PRV XJ infection led to a significant increase in the IL-6 and TNF-α expression in serum at 3 dpi, whereas the IL-6 and TNF-α expression in rPRV-NC56 and rPRV XJ-ΔgE/gI/TK-EGFP infected groups had no significant differences (Figures 5B,C). Meanwhile we analyzed the pathological changes in the brain tissues of mice in each group. The results of pathological analysis showed that PRV XJ infection led to a disordered arrangement of cell layers in the cerebral cortex and degeneration and necrosis of neuronal cells, but the rPRV-NC56 and rPRV XJ-ΔgE/gI/TK-EGFP infection groups had no pathological changes (Figure 5D). The results showed that rPRV-NC56 virus is safe for mice.

### The Recombinant Pseudorabies Virus-NC56 Can Colonize and Express the Target Protein in the Lung of Mice for a Long Time

**FIGURE 6:**
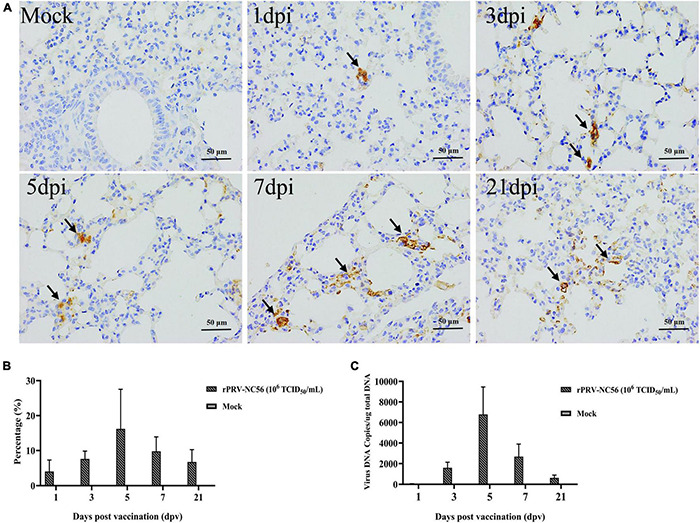
The colonization properties of rPRV-NC56 in mouse lungs. **(A)** IHC identification of GP5 protein expression in the mouse lungs of rPRV-NC56-infected mice at 1, 3, 5, 7, and 21 dpv. The brown precipitate indicated by the black arrow indicates the cells expressing GP5 protein (×400). **(B)** Percentage of positive cells expressing GP5 protein identified by IHC. **(C)** qPCR identification of gB gene in the lungs of rPRV-NC56-immunized mice.

To investigate the proliferation and the expression of target gene in the lungs of rPRV-NC56-immunized mice, we identified the colonization and target protein expression in the lungs of mice immunized with rPRV-NC56 at different times by qPCR and IHC, respectively. The results of IHC detection are shown in Figures 6A,B. A few GP5 IHC-positive cells could be found in the lung at 1 dpv after rPRV-NC56 i.m. immunization; the number of positive cells gradually increased at 3 dpv, the most at 5 dpv, and the positive cells could still be found in the lung until 21 dpv. The viral genome DNA in the lung was detected by qPCR. The calibration equation is Y = −3.295X + 33.868, E = 97.6%, *R*^2^ = 1.000, Slope = −33.382, y-int = 33.868. In the results shown in Figure 6C, gB gene could be detected in the lung at 1 dpv after rPRV-NC56 immunization, which lasted until the 21st day, and the copies of gB gene was the highest at 5 dpv.

### NADC30-Like Porcine Reproductive and Respiratory Syndrome Virus-Specific Antibodies and Pseudorabies Virus-Specific gB Antibodies Can Be Induced by Recombinant Pseudorabies Virus-NC56 in Mice

**FIGURE 7:**
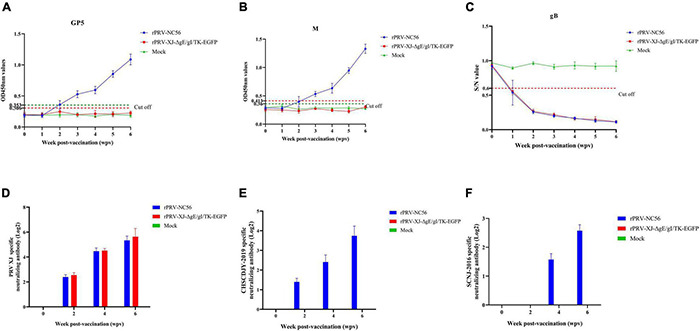
Immune responses after immunization with rPRV-NC56. **(A)** The GP5-specific antibodies were calculated by ELISA. **(B)** The M-specific antibodies were calculated by ELISA. **(C)** The gB-specific antibodies were calculated by ELISA. **(D)** Detection results of anti-PRV XJ-neutralizing antibody titer in the serum of immunized mice. **(E)** Detection results of anti-PRRSV CHSCDJY-2019-neutralizing antibody titer in the serum of immunized mice. **(F)** Detection results of anti-PRRSV SCNJ-2016-neutralizing antibody titer in the serum of immunized mice.

To evaluate the specific antibody response to rPRV-NC56, indirect ELISA assays and specific virus-neutralizing antibody (VNA) test were used to detect antigen-specific antibodies and neutralizing antibody, and the results are shown in Figure 7. The results showed that low antibodies were detected in all vaccinated groups after 2 wpv for the primary vaccination, and the antibodies to gB and GP5, and M turned positive after 1 wpv for the booster vaccination. The virus-specific antibody levels (OD450 values) were gradually increased over time after vaccination. The GP5- and M-specific antibodies were not detected in the control and rPRV XJ-ΔgE/gI/TK-EGFP-immunized mouse group (Figures 7A,B). All mouse groups vaccinated with rPRV-NC56 and rPRV XJ-ΔgE/gI/TK-EGFP contained similar levels of gB antibodies in the postimmunization serum (Figure 7C). The rPRV-NC56 and rPRV XJ-ΔgE/gI/TK-EGFP vaccinated group exhibited similar neutralization titers against PRV XJ (*p* = 0.14), and the VNA titers of the control group was lower than 1:2 (Figure 7D). The rPRV-NC56 vaccinated group exhibited higher levels of VNA titers against PRRSV CHSCDJY-2019 than SCNJ-2016 (Figures 7E,F). This result indicates that rPRV-NC56 induces humoral immunity specific for PRRSV GP5 and M protein and does not affect viral production PRV gB antibodies.

### The Innate Immune Cytokines Were Induced by Recombinant Pseudorabies Virus-NC56 in Mice

**FIGURE 8:**
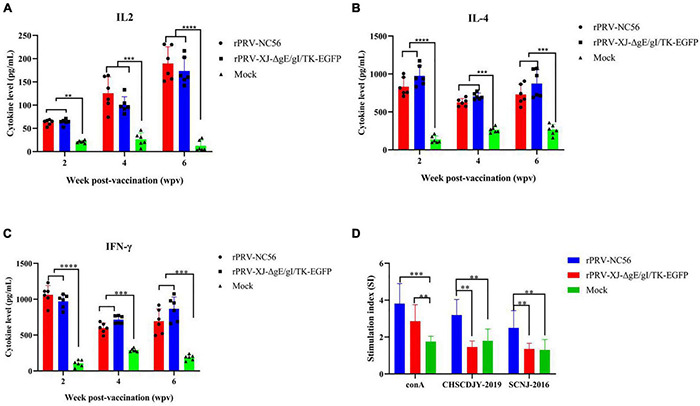
The T cellular response analyses of blood sample from rPRV XJ-ΔgE/gI/TK-EGFP, rPRV-NC56, and nonvaccinated groups. **(A)** Plasma IL-2 was detected by ELISA. **(B)** Plasma IL-4 was detected by ELISA. **(C)** Plasma IFN-γ was detected by ELISA. **(D)** Splenic lymphocytes were assayed for T-cell-specific proliferative responses using the MTT kit. *p < 0.05; ^**^p < 0.01; ^***^p < 0.005; and ^****^p < 0.001.

Potent induction of adaptive immunity depends on efficient activation of the innate immune system. Cytokines, such as IFN-γ, IL-2, IL-4, and IL-6, can enhance adaptive immune response and play an important role in regulating innate and adaptive immunity (Striz et al., 2014). IL-2, IL-4, and IFN-γ were measured by ELISA in the peripheral blood of each mouse group. The rPRV-NC56- and rPRV XJ-ΔgE/gI/TK-EGFP-immunized mice compared with the control group showed significant upregulation of IL-2, IL-4, and IFN-γ (Figure 8). IL-2 in the peripheral blood of rPRV-NC56- and rPRV XJ-ΔgE/gI/TK-EGFP-immunized mice at 2, 4, and 6 wpv was continuously increased after primary vaccination, and IL-4 and IFN-γ in the peripheral blood of rPRV-NC56- and rPRV XJ-ΔgE/gI/TK-EGFP-immunized mice were the highest (Figures 8A–C). The majority of T cells produce IL-2, IL-4, and IFN-γ during robust T-cell responses.

### The Specific T-Cell Immune Responses Were Induced by Recombinant Pseudorabies Virus-NC56 in Mice

**FIGURE 9:**
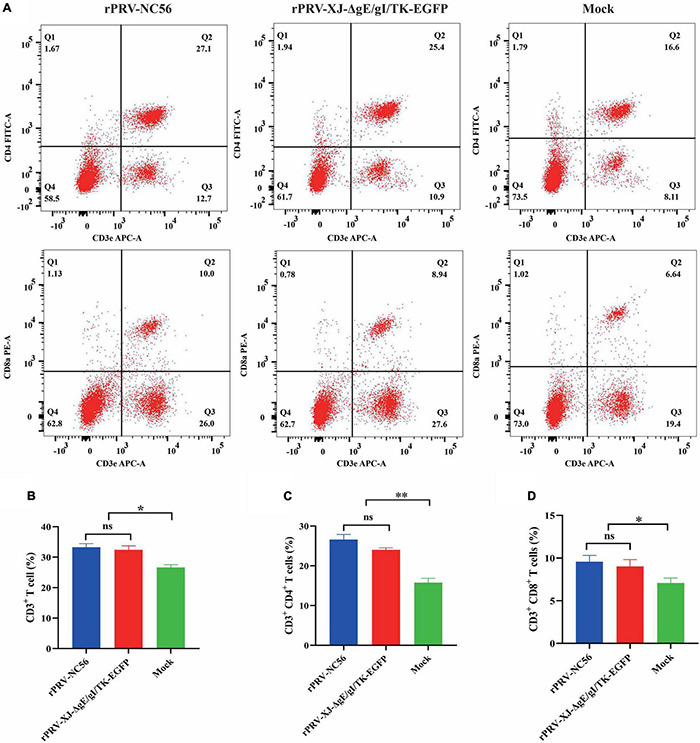
Flow cytometric analysis of CD3^+^/CD4^+^/CD8^+^ cells in the spleen of rPRV XJ-ΔgE/gI/TK-EGFP, rPRV-NC56, and nonvaccinated groups. **(A)** Flow cytometry analysis of CD3^+^, CD3^+^ CD4^+^, and CD3^+^ CD8^+^ T-cell populations at 2 weeks postvaccination (wpv) after immunization (*n* = 6/each group). The data show the percentage of CD3^+^ **(B)**, CD3^+^ CD4^+^ **(C)**, and CD3^+^ CD8^+^ **(D)** T cells in the total splenocytes. By GraphPad Prism 5.0, unpaired *t*-test, *p < 0.05; ^**^p < 0.01; ^***^p < 0.005; and ^****^p < 0.001.

We detected the activation of lymphocytes in spleen and the proliferation of splenocytes stimulated by target antigen to investigate the cellular responses induced by rPRV-NC56 immunization of mice. The splenocytes of rPRV-NC56-immunized mice increased significantly after being stimulated by inactivated PRRSV CHSCDJY-2019 and SCNJ-2016 as stimulants, but CHSCDJY-2019 could cause a stronger proliferative response than SCNJ-2016 (Figure 8D). The percentage of CD3^+^, CD4^+^, and CD8^+^ T-cell subpopulations in splenic were detected by flow cytometry at 4 wpv. The CD3^+^, CD4^+^ and CD8^+^ T-cell ratios were different between the three groups (Figure 9). The CD3^+^, CD8^+^, and CD4^+^ T-cell ratios of rPRV-NC56- and rPRV XJ-ΔgE/gI/TK-EGFP-immunized mouse group had no significant difference, but they were significantly higher than those in the control group (*p* < 0.05 and *p* < 0.01).

### The Recombinant Pseudorabies Virus-NC56 Strains Can Protect Mice 100% From Challenge by the Virulent Strain

**FIGURE 10:**
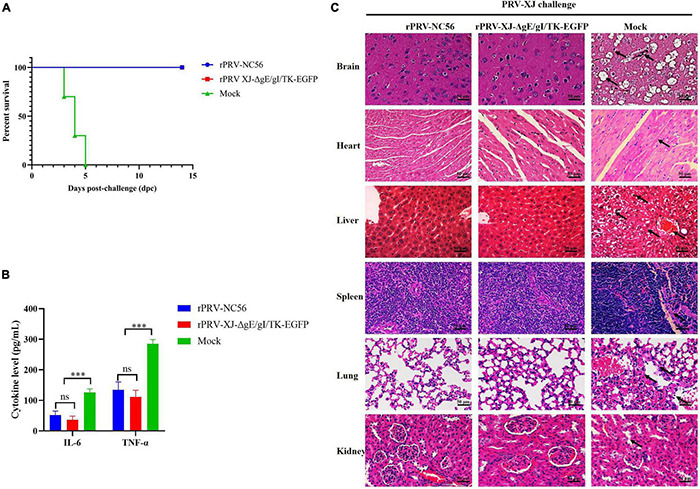
Recombinant pseudorabies virus-NC56 has a 100% protective capacity against challenge by virulent PRV XJ. **(A)** The survival rate of rPRV XJ-ΔgE/gI/TK-EGFP, rPRV-NC56, and nonvaccinated mice groups challenged with PRV XJ strain. **(B)** The results of inflammatory cytokines (IL-6 and TNF-α) in the serum of mice in each group at 3 dpc by ELISA. **(C)** Histopathological examination of mice brain, heart, liver, spleen, lungs, and kidneys at 5 days postchallenge (dpc) (×400). *p < 0.05; ^**^p < 0.01; ^***^p < 0.005; and ^****^p < 0.001. Pathological lesions in all tissues were described in the results.

To further investigate the protective immunity of rPRV-NC56, after 4 weeks of booster vaccination, the immunized mice were challenged with 1 × 10^3.74^ TCID_50_ of PRV XJ by intramuscular injection. All mice were observed for 14 dpc. During the observation period, no clinical symptoms related to pseudorabies were observed in mice immunized with RPRV-NC56 and rPRV XJ-ΔgE/gI/TK-EGFP, and no mice died (Figure 10A). The mice in the control group began to die at 3 dpc, and all mice died at 5 dpc. At 3 dpc, the blood of all challenged mice was collected from the tail vein to detect the inflammatory cytokines IL-6 and TNF-α. There was a highly significant upregulation of IL-6 and TNF-α in the control group compared with the rPRV-NC56, rPRV XJ-ΔgE/gI/TK-EGFP immune group, while there was no significance in the rPRV-NC56 and rPRV XJ-ΔgE/gI/TK-EGFP immune group (Figure 10B).

Histopathological examination of mice brain, heart, liver, spleen, lungs, and kidneys were performed at 5 dpc. The rPRV-NC56- and rPRV XJ-ΔgE/gI/TK-EGFP-vaccinated group did not display significant histopathological changes in the tissues. However, in the control group, there were neuronal cell degeneration, necrosis, and vacuolization in the cortical region of the brain, hemorrhage in the heart and liver, vacuolar degeneration and necrosis of the liver cells, unclear demarcation of white pulp and red pulp and thickening of the trabecular meshwork in the spleen, thickening of the alveolar wall, degeneration and necrosis of some alveolar epithelial cells and inflammatory cell infiltration in the lung interstitium in the lung, glomerular edema, and cell necrosis in the kidneys (Figure 10C).

## Discussion

Porcine reproductive and respiratory syndrome remains a major challenge in the pig industry and causes significant economic loss annually. Vaccine immunity remains an important means to control the PRRSV outbreak and epidemic. The continuous evolution of PRRSV, resulting in simultaneous outbreaks and epidemics of multisubtype strains, has made PRRS prevention and control more complicated. The NADC30-like PRRSV strain is the main epidemic strain with the second detection rate compared with the HP-PRRSV strain in China (Sun et al., 2020). Whether the currently available commercial PRRSV vaccine based on classical PRRSV and HP-PRRSV strains can prevent or worsen the diseases infected by the new subtype PRRSV strains requires to be further confirmed. Several studies have reported the evaluation of the protective effect of commercial PRRSV vaccine on NADC30-like PRRSV strain. Ingelvac PRRS MLV (United States) and modified live virus (MLV) vaccine derived from classical PRRSV (VR2332) or HP-PRRSV (TJ and JXA1) have been reported to provide optimistic cross protection for some NADC30-like PRRSV strains isolated in China (Zhang et al., 2018; Wei et al., 2019; Chai et al., 2020). However, some studies have shown that the above commercial vaccines can only provide limited protection against some NADC30 PRRSV strains (FJWQ16, HNhx, and CHsx1401) (Bai et al., 2016; Zhou et al., 2017; Wei et al., 2019; Chen et al., 2021). PRRSV CHSCDJY-2019 was isolated from the serum of piglets with severe respiratory clinical symptoms and viremia. It was the result of the recombination of NADC30 strain with TJ-like PRRSV and HP-PRRSV (Zhao et al., 2021). GP5 is the major glycoprotein of PRRSV and carries the major neutralizing epitope (Popescu et al., 2017). M is the most conserved membrane protein in PRRSV, which can cause strong cellular immune response (Bautista et al., 1999; Murtaugh et al., 2010). The formation of disulfide-linked complexes between GP5 and M through disulfide bonds is the key mechanism for the production of VLPs (Mardassi et al., 1996). Fusion expression of GP5 and M proteins or multigene co-expression can cause more neutralizing antibodies against GP5 than GP5 protein alone (Jiang et al., 2006, 2007; Zheng et al., 2007).

The expression of foreign genes by gene deletion-attenuated PRV live virus vector is a very mature antigen presentation system. In previous studies, we have confirmed that piglets immunized with JXA1-R vaccine will still have elevated body temperature, respiratory symptoms, severe viremia, and lung pathological changes after being infected with CHSCDJY-2019 (data not published). In the present study, we constructed a recombinant pseudorabies virus (rPRV-NC56) co-expressing GP5 and M proteins of NADC30-like PRRSV. The gE, gI, and TK genes of the recombinant virus were knocked out at the same time as foreign gene insertion. In order to enable GP5 and M proteins to express independently without affecting each other’s later modification, self-cleavage T2A and P2A peptides were introduced (Tang X. et al., 2016). The rPRV-NC56 carries the foreign gene to proliferate within BHK-21 cells for 21 generations. This strain is a potential marker vaccine for PRRSV DIVA and a targeted vaccine against NADC30-like PRRSV strain. In addition, PRV gE, gI, and TK gene-deficient live attenuated vaccine can colonize the host for a long time and cause a strong immune response (Hu et al., 2015; Wang et al., 2020). Therefore, we anticipate that the foreign gene would be persistently expressed, together with PRV replication, to constantly stimulate immune responses in the host. In addition, several studies have confirmed that PRRSV GP5 and M proteins can assemble into VLPs, and independently expressed GP5 and M proteins are capable of self-assembling intracellular assembly into VLPs (Binjawadagi et al., 2016; García Durán et al., 2016; Xu et al., 2021). PRRSV VLPs can induce a stronger immune response than commercial vaccines (Nam et al., 2013). In this study, we found that the virus particles of pseudorabies virus and VLPs of PRRSV in BHK-21 cells were infected with rPRV-NC56. The morphology and size of PRRSV VLPs (approximately 40–60 nm) expressed by rPRV-NC56 were largely similar to PRRSV virions and PRRSV VLPs composed of GP5 and M proteins expressed by baculovirus (Nam et al., 2013; Binjawadagi et al., 2016; Xu et al., 2021). We detected the Viral gene copies and the number of infection-positive cells in the lung tissue of rPRV-NC56-immunized mice by qRT-PCR and IHC. The rPRV-NC56 can colonize and express foreign protein in lung tissue for a long time in mice. From 3 to 21 dpv after immunization, the gB gene of rPRV-NC56 can be detected in the lungs, and IHC can detect the sustained expression of GP5 protein in the lungs.

Mouse is the natural host of PRV and the research model for PRV vaccine development and neural transmission. We evaluated the safety and immunogenicity of rPRV-NC56 in mice. The inflammatory cytokines IL-6 and TNF-α in the serum of high-dose rPRV-NC56-immunized mice did not change significantly, while they were significantly upregulated in the serum of PRV XJ-infected mice. No pathological changes were found in the brain of mice immunized with high-dose rPRV-NC56, compared with PRV XJ-infected mice. This is consistent with previous studies, indicating that the NADC30-like PRRSV/PRV bivalent vaccine is safe and will not cause clinical symptoms and histopathological changes in mice (Zhao et al., 2020). After the second immunization, specific antibodies and neutralizing antibodies against PRV and PRRSV in mice inoculated with rPRV-NC56 increased continuously. The neutralizing antibody titer of NADC30-like PRRSV in the serum of rPRV-NC56-immunized mice was higher than that of HP-PRRSV. PRRSV infection has been reported to decrease in CD4^+^ T cells and increase in CD8^+^ T cells in piglets (Tingstedt and Nielsen, 2004; Dwivedi et al., 2012). In this study, rPRV-NC56 could effectively activate the specific T-cell immune system, and CD3^+^ and CD3^+^ CD8^+^ T cells increased significantly after inoculation with attenuated strain compared with other groups. However, there was no significant difference in the CD4^+^ T cell level in the experimental and control groups. This may also have been an association between the PRRSV GP5 and M proteins expressed by rPRV-NC56.

The stimulation of T-cell-mediated IFN-γ response upon immunization is a major focus for assessing the improvement of vaccine efficacy. There is a positive correlation between the frequency of virus-specific IFN-γ-secreting cells and the clinical outcome upon PRRSV infection after immunization with the PRRSV vaccine (Martelli et al., 2009). Meanwhile, IFN-γ plays a key role in innate and adaptive immunity induced by herpes simplex virus and PRV (Paludan et al., 2002; Yao et al., 2007).

We investigated the proliferative response of rPRV-NC56-immunized mice spleen cells stimulated by NADC30-like PRRSV and inactivated HP-PRRSV. The stimulation index and IFN-γ concentrations in the cell culture medium of the inactivated NADC30-like PRRSV stimulation group were significantly higher than that of the inactivated HP-PRRSV stimulation group, indicating that there was a significant difference in cell response between the two subtypes of PRRSVs. The concentrations of IL-2, IL-4, and IFN-γ in the sera of rPRV-NC56- and rPRV XJ-ΔgE/gI/TK-EGFP-immunized mice were similar, but significantly upregulated compared with the nonimmunized group. IL-2 is produced by T cells, which are capable of promoting killer cell differentiation and effector, B-cell proliferative differentiation, and macrophage activation. IL-4 is produced by Th2 subsets of cells, promoting proliferation and differentiation of B cells and enhance the humoral immune response.

## Conclusion

In conclusion, we constructed a recombinant pseudorabies virus (rPRV-NC56) co-expressing NADC30-like PRRSV GP5 and M proteins. The GP5 and M proteins were autonomously loaded into VLPs in cells. The rPRV-NC56 colonizes the lung tissue of mice for prolonged periods and continuously expresses NADC30-like PRRSV GP5 and M proteins. The rPRV-NC56, similar to rPRV XJ-ΔgE/gI/TK-EGFP, could effectively activate protective immunity and produce specific antibodies. The rPRV-NC56 could activate high levels of neutralizing antibodies against PRV and NADC30-like PRRSV. Vaccination and challenge experiments showed that rPRV-NC56 elicited complete protection of mice against PRV. The rPRV-NC56 vaccine could serve as a DIVA vaccine in the further control and eradication of PRRSV. The evaluation of the safety and immunogenicity of rPRV-NC56 based on the PRV natural host mouse model can provide a reference for rPRV-NC56 in conducting vaccine evaluation studies on animals.

## Data Availability Statement

The original contributions presented in the study are included in the article/Supplementary Material, further inquiries can be directed to the corresponding author.

## Ethics Statement

The animal study was reviewed and approved by the Sichuan Provincial Laboratory Animal Management Committee [License No: SYXK (chuan) 2019-187].

## Author Contributions

ZX and LZ conceived the project. JZ designed the experiments and wrote the manuscript. JZ, FL, LX, and YZ performed most of the experiments. XS, HD, SG, and YH contributed the materials and participated in the discussion. ZX supervised the work and edited the final version of the manuscript, which was read and approved by all authors.

## Conflict of Interest

The authors declare that the research was conducted in the absence of any commercial or financial relationships that could be construed as a potential conflict of interest.

## Publisher’s Note

All claims expressed in this article are solely those of the authors and do not necessarily represent those of their affiliated organizations, or those of the publisher, the editors and the reviewers. Any product that may be evaluated in this article, or claim that may be made by its manufacturer, is not guaranteed or endorsed by the publisher.
