# Supplementary material for: The Construction and Immunogenicity Analyses of Recombinant Pseudorabies Virus With NADC30-Like Porcine Reproductive and Respiratory Syndrome Virus-Like Particles Co-expression
Source: Front Microbiol. 2022 Mar 2;13:846079. doi: 10.3389/fmicb.2022.846079 (PMC8924499; doi:10.3389/fmicb.2022.846079)
Supplement: Supplementary file 1 [file Data_Sheet_1.docx]

**Supplementary Materials**

Table S1. Oligonucleotide primers used in this study.

| Target gene | Primers | Primer sequences (5’-3’) | Amplicon length (bp) | Application |
| --- | --- | --- | --- | --- |
| PRRSV-ORF5 | PRRSV-ORF5-F | 5*GCCACGAACTTCTCTCTGTTAAAGCAAGCAGGAGACGTGGAAGAAAACCCCGGTCCT*atgttggggaagtgcttgac | 660 | For construction of pEGFP-2A-NC-OFR5-6 |
|  | PRRSV-ORF5-R | 5′-tcagagacgaccccatagtt-3′ |  |  |
| PRRSV-M | PRRSV-ORF6-F | *gcatggacgagctgtacaagtccgga*ggcagtggaggcagtggaGAGGGCAGAGGAAGTCTGCTAACATGCGGTGACGTCGAGGAGAATCCTGGCCCAgaattcatggggtcgtctctagacgacttctgc | 683 |  |
|  | PRRSV-ORF6-R | *AGGACCGGGGTTTTCTTCCACGTCTCCTGCTTGCTTTAACAGAGAGAAGTTCGTGGC*ggcagaacctttggcatatttaacaaggtttaccacc |  |  |
| PRV-gE | PRV-gE-F | ATCTGGACGTTCCTGCCC | 534 | rPRV-NC56  verification |
|  | PRV-gE-R | GTAGATGCAGGGCTCGTACA |  |  |
| PRV-gB | PRV-gB-F | AGGGACCGCTTCTACGTCT | 464 |  |
|  | PRV-gB-R | TGTAGGTGTCGTTGGTGGTG |  |  |
| PRV-gI | PRV-gI-F | TCGCCGAGCAACTACAGCGG | 589 |  |
|  | PRV-gI-R | CGGCGTCGTCGTCTCCGCGT |  |  |
| PRV-TK（detection） | PRV-TK-F1 | GCTTCATCGTCGGGGACATC | 604 |  |
|  | PRV-TK-R1 | GAGGGTCACACCCCCATCTC |  |  |
| PRV-TK  (complete CDS) | PRV-TK-F | GATGACATACACATGGCTTTATACGCGCC | 1579 |  |
|  | PRV-TK-R | TCACCGCCGCGGCCCGGCGACGTACTC |  |  |

**Figure S1. Sequence of pEGFP-2A-NC-OFR5-6 and plasmid map.**


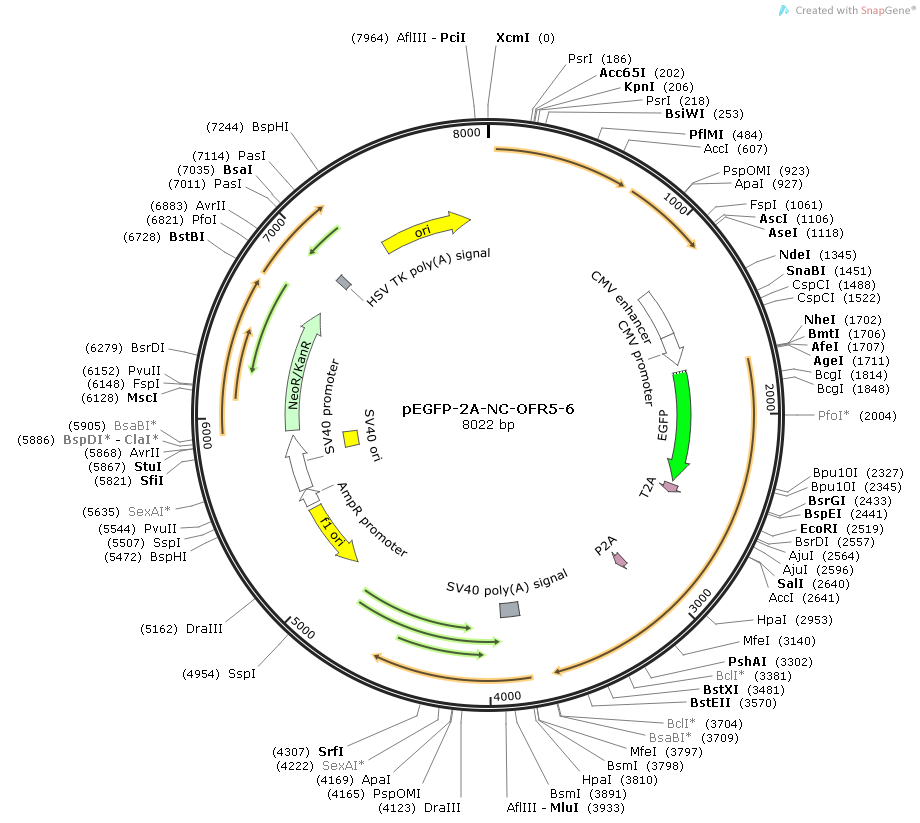


TAGTTGGCGTGAACATCCTCACCGACTTCATGGTGGCGCTCCCCGAGGGGCAAGAGTGCCCGTTCGCCCGCGTGGACCAGCACCGCACGTACAAGTTCGGCGCGTGCTGGAGCGACGACAGCTTCAAGCGGGGCGTGGACGTGATGCGATTCCTGACGCCGTTCTACCAGCAGCCCCCGCACCGGGAGGTGGTGAACTACTGGTACCGCAAGAACGGCCGGACGCTCCCGCGGGCCTACGCCGCCGCCACGCCGTACGCCATCGACCCCGCGCGGCCCTCGGCGGGCTCGCCGAGGCCCAGGCCCCGGCCCCGGCCCAGGCCCCGGCCGAAGCCCGAGCCCGCCCCGGCGACGCCCGCGCCCCCCGGCCGCCTGCCCGAGCCGGCGACGCGGGACCACGCCGCCGGGGGGCGCCCCACGCCGCGACCCCCGAGGCCCGAGACGCCGCACCGCCCCTTCGCCCCGCCGGCCGTCGTGCCCAGCGGGTGGCCGCAGCCCGCGGAGCCGTTCCCGCCCCGGACCACCGCCGCGCCGGGCGTCTCGCGCCACCGCTCGGTGATCGTCGGCACGGGCACCGCGATGGGCGCGCTCCTGGTGGGCGTGTGCGTCTACATCTTCTTCCGCCTGAGGGGGGCGAAGGGGTATCGCCTCCTGGGCGGTCCCGCGGACGCCGACGAGCTAAAAGCGCAGCCCGGTCCGTAGCCTCCGCAGTACCGGCGTCGATGATGATGGTGGCGCGCGACGTGACCCGGCTCCCCGCGGGGCTCCTCCTCGCCGCCCTGACCCTGGCCGCCCTGACCCCGCGCGTCGGGGGCGTCCTCTTCAGGGGCGCCGGCGTCAGCGTGCACGTCGCCGGCAGCGCCGTCCTCGTGCCCGGCGACGCGCCCAACCTGACGATAGACGGGACGCTGCTGTTTCTGGAGGGGCCCTCGCCGAGCAACTACAGCGGGCGCGTGGAGCTGCTGCGCCTCGACCCCAAGCGCGCCTGCTACACGCGCGAGTACGCCGCCGAGTACGACCTCTGCCCCCGCGTGCACCACGAAGCCTTCCGCGGCTGCCTGCGCAAGCGCGAGCCGCTCGCCCGGCGCGCGTCCGCCGCGGTGGAGGCGCGCCGGCTATTAATAGTAATCAATTACGGGGTCATTAGTTCATAGCCCATATATGGAGTTCCGCGTTACATAACTTACGGTAAATGGCCCGCCTGGCTGACCGCCCAACGACCCCCGCCCATTGACGTCAATAATGACGTATGTTCCCATAGTAACGCCAATAGGGACTTTCCATTGACGTCAATGGGTGGAGTATTTACGGTAAACTGCCCACTTGGCAGTACATCAAGTGTATCATATGCCAAGTACGCCCCCTATTGACGTCAATGACGGTAAATGGCCCGCCTGGCATTATGCCCAGTACATGACCTTATGGGACTTTCCTACTTGGCAGTACATCTACGTATTAGTCATCGCTATTACCATGGTGATGCGGTTTTGGCAGTACATCAATGGGCGTGGATAGCGGTTTGACTCACGGGGATTTCCAAGTCTCCACCCCATTGACGTCAATGGGAGTTTGTTTTGGCACCAAAATCAACGGGACTTTCCAAAATGTCGTAACAACTCCGCCCCATTGACGCAAATGGGCGGTAGGCGTGTACGGTGGGAGGTCTATATAAGCAGAGCTGGTTTAGTGAACCGTCAGATCCGCTAGCGCTACCGGTCGCCACCATGGTGAGCAAGGGCGAGGAGCTGTTCACCGGGGTGGTGCCCATCCTGGTCGAGCTGGACGGCGACGTAAACGGCCACAAGTTCAGCGTGTCCGGCGAGGGCGAGGGCGATGCCACCTACGGCAAGCTGACCCTGAAGTTCATCTGCACCACCGGCAAGCTGCCCGTGCCCTGGCCCACCCTCGTGACCACCCTGACCTACGGCGTGCAGTGCTTCAGCCGCTACCCCGACCACATGAAGCAGCACGACTTCTTCAAGTCCGCCATGCCCGAAGGCTACGTCCAGGAGCGCACCATCTTCTTCAAGGACGACGGCAACTACAAGACCCGCGCCGAGGTGAAGTTCGAGGGCGACACCCTGGTGAACCGCATCGAGCTGAAGGGCATCGACTTCAAGGAGGACGGCAACATCCTGGGGCACAAGCTGGAGTACAACTACAACAGCCACAACGTCTATATCATGGCCGACAAGCAGAAGAACGGCATCAAGGTGAACTTCAAGATCCGCCACAACATCGAGGACGGCAGCGTGCAGCTCGCCGACCACTACCAGCAGAACACCCCCATCGGCGACGGCCCCGTGCTGCTGCCCGACAACCACTACCTGAGCACCCAGTCCGCCCTGAGCAAAGACCCCAACGAGAAGCGCGATCACATGGTCCTGCTGGAGTTCGTGACCGCCGCCGGGATCACTCTCGGCATGGACGAGCTGTACAAGTCCGGAGGCAGTGGAGGCAGTGGAGAGGGCAGAGGAAGTCTGCTAACATGCGGTGACGTCGAGGAGAATCCTGGCCCAGAATTCATGGGGTCGTCCATAGACGACTTCTGCAATGATAGCACGGCTGTGCAGAAGGTGCTATTGGCGTTTTCTATCACCTACACGCCAATAATGATATATGCCTTAAAAGTGAGTCGCGGTCGACTGCTGGGGCTCTTGCACCTTTTAATTTTCCTGAATTGTGCTTTCACTTTTGGGTATATGACATTTGTTCATTTTCAGAGTACAAACAGGGTTGCACTTACCTTGGGTGCAGTAGTCGCTCTCCTCTGGGGGGTGTATTCAGCCATGGAAATCTGGAGATTCATCACCTCCAGATGCCGGTTGTGCTTGCTAGGCCGCAGGTACATTCTGGCCCCTGCCCACCACGTTGAAAGTGCCGCAGGCTTTCATCCGATAACGGCAAGTGATAACCACGCATTTGTCGTCCGGCGTCCCGGCTCCACTACGGTTAACGGCACACTGGTGCCAGGGTTGAAAAGCCTCGTGTTGGGTGGCAGAAGAGCTGTTAAACGAGGAGTGGTGAACCTTGTTAAATATGCCAAAGCCACGAACTTCTCTCTGTTAAAGCAAGCAGGAGACGTGGAAGAAAACCCCGGTCCTATGTTGGAGAAATGCTTGACCGCGGGTTATTGCTCGCAATTGCCTTTTTTGTGGTGTATCGTGCCATTCTGTTTTGCTGTGCTCGTCAACGCCAACAGCAACAGCAGCTCCCATCTACAGTTGATTTATAACCTGACGATATGTGAGCTGAATGGCACAGATTGGCTGAACGAAAGATTTTACTGGGCAGTGGAGACTTTCGTCATCTTTCCTGTACTGACTCATATTGTCTCTTACGGAGCCCTTACCACTAGCCATTTTCTTGACACGGTCGGCCTGATCACTGTGTCCACCGCCGGTTATCTTCACGGGCGGTATGTATTAAGCAGCATCTATGCTGTCTGTGCCCTGGCTGCATTTGCTTGCTTCGCCATTAGGTTGGCGAAAAATTGCATGTCCTGGCGCTACTCATGCACCAGATACACTAATTTCCTTCTGGATACTAAGGGCAAACTCTACCGCTGGCGGTCACCCGTCATCATAGAGAAAGGGGGTAAAGTTGACGTTGGGGGTCATTTGATTGACCTCAAGAGAGTTGTGCTTGATGGTTCCGCGGCAACCCCTGTAACCAAGATTTCAGCGGAACAATGGGGTCGTCCATGATCATAATCAGCCATACCACATTTGTAGAGGTTTTACTTGCTTTAAAAAACCTCCCACACCTCCCCCTGAACCTGAAACATAAAATGAATGCAATTGTTGTTGTTAACTTGTTTATTGCAGCTTATAATGGTTACAAATAAAGCAATAGCATCACAAATTTCACAAATAAAGCATTTTTTTCACTGCATTCTAGTTGTGGTTTGTCCAAACTCATCAATGTATCTTAACGCGTATCTCCCCCGGCTCGCTGGCCCTGCTGCCGCGCGCCGTGCGCCCCGTCGTGCGGACGCGGTCCGACCCCACGGCGCCGTTCTACATCACCACCGAGACGCACGAGCTGACGCGGCGCCCCCCGGCGGACGGCTCGAAGCCCGGGGAGCCCCTCAGGATCAGCCCACCCCCGCGGCTGGACACGGAGTGGTCGTCCGTCCTGAACGGGATCCAGTACCTGAACTCGGGGGCCCGGGGCACGGCCCCCGTCCACCTGTGGATCCTGGGCGCCGCCGACCTCTGCGACCAGGTGCTCCTGGCCGCCTCCCGCAGCACCGCCGCCGGAGCCTCCCACGCCCAGACGGGCGCGCGCCTGACCCGGCGCCGGCCCGGGCTGACGGACGCCGACGCCCTGGACGTGATCGTCGCCGGGATCCAGGCGACCCGCGCCATGTTCGCGCGGGTCCACAACCGCTCCTGGCGCCACGCCGGCGAGTGGACGGAGGCCCTGCACTCCCAGATCGTGACCCGGGGCGACGTGCGCCGGCGCCGAGGCGGGCGCGGCAACGGACGCGAGCGCGCCCCGCGATGTACCATCTCCTAGACGGCAGGATCTCTCCGCGTCCCCCACCCCCCCAAAAAACAAACAATAAACGCTCTCGCTCTGGCACCCGATGACACGCCTCCGTCCTCTCTCTCCCTCCCACTGACGCCACCCCTCCCCTCGCCGACAACGCCATCGTCGCCCGGCGTCGGCCGGACCGGCGGTTCTCCCCCCACCCCGTCCCCCCCCACCCCGTCCCCCCCCACCCCTGCCCCCGCTTCGTCCGACTCTCGCCCCCCGCGGGAGGGTTCCGCGGCTCGCTCCCCGTCTCATCCCCCCGTCTCATCCCCCCGTCTCACTCCCATCTCCCTCCCTCCACCCCGTCTCATCCCCCCATCTCCCTTCCCCACGAGGGCCGGGAGGGGAAAAAACGCCCGAGAGACGAGAGAGTTGAGGTTCGAGCGGCGGGCCGCCGTGAAATTGTAAGCGTTAATATTTTGTTAAAATTCGCGTTAAATTTTTGTTAAATCAGCTCATTTTTTAACCAATAGGCCGAAATCGGCAAAATCCCTTATAAATCAAAAGAATAGACCGAGATAGGGTTGAGTGTTGTTCCAGTTTGGAACAAGAGTCCACTATTAAAGAACGTGGACTCCAACGTCAAAGGGCGAAAAACCGTCTATCAGGGCGATGGCCCACTACGTGAACCATCACCCTAATCAAGTTTTTTGGGGTCGAGGTGCCGTAAAGCACTAAATCGGAACCCTAAAGGGAGCCCCCGATTTAGAGCTTGACGGGGAAAGCCGGCGAACGTGGCGAGAAAGGAAGGGAAGAAAGCGAAAGGAGCGGGCGCTAGGGCGCTGGCAAGTGTAGCGGTCACGCTGCGCGTAACCACCACACCCGCCGCGCTTAATGCGCCGCTACAGGGCGCGTCAGGTGGCACTTTTCGGGGAAATGTGCGCGGAACCCCTATTTGTTTATTTTTCTAAATACATTCAAATATGTATCCGCTCATGAGACAATAACCCTGATAAATGCTTCAATAATATTGAAAAAGGAAGAGTCCTGAGGCGGAAAGAACCAGCTGTGGAATGTGTGTCAGTTAGGGTGTGGAAAGTCCCCAGGCTCCCCAGCAGGCAGAAGTATGCAAAGCATGCATCTCAATTAGTCAGCAACCAGGTGTGGAAAGTCCCCAGGCTCCCCAGCAGGCAGAAGTATGCAAAGCATGCATCTCAATTAGTCAGCAACCATAGTCCCGCCCCTAACTCCGCCCATCCCGCCCCTAACTCCGCCCAGTTCCGCCCATTCTCCGCCCCATGGCTGACTAATTTTTTTTATTTATGCAGAGGCCGAGGCCGCCTCGGCCTCTGAGCTATTCCAGAAGTAGTGAGGAGGCTTTTTTGGAGGCCTAGGCTTTTGCAAAGATCGATCAAGAGACAGGATGAGGATCGTTTCGCATGATTGAACAAGATGGATTGCACGCAGGTTCTCCGGCCGCTTGGGTGGAGAGGCTATTCGGCTATGACTGGGCACAACAGACAATCGGCTGCTCTGATGCCGCCGTGTTCCGGCTGTCAGCGCAGGGGCGCCCGGTTCTTTTTGTCAAGACCGACCTGTCCGGTGCCCTGAATGAACTGCAAGACGAGGCAGCGCGGCTATCGTGGCTGGCCACGACGGGCGTTCCTTGCGCAGCTGTGCTCGACGTTGTCACTGAAGCGGGAAGGGACTGGCTGCTATTGGGCGAAGTGCCGGGGCAGGATCTCCTGTCATCTCACCTTGCTCCTGCCGAGAAAGTATCCATCATGGCTGATGCAATGCGGCGGCTGCATACGCTTGATCCGGCTACCTGCCCATTCGACCACCAAGCGAAACATCGCATCGAGCGAGCACGTACTCGGATGGAAGCCGGTCTTGTCGATCAGGATGATCTGGACGAAGAGCATCAGGGGCTCGCGCCAGCCGAACTGTTCGCCAGGCTCAAGGCGAGCATGCCCGACGGCGAGGATCTCGTCGTGACCCATGGCGATGCCTGCTTGCCGAATATCATGGTGGAAAATGGCCGCTTTTCTGGATTCATCGACTGTGGCCGGCTGGGTGTGGCGGACCGCTATCAGGACATAGCGTTGGCTACCCGTGATATTGCTGAAGAGCTTGGCGGCGAATGGGCTGACCGCTTCCTCGTGCTTTACGGTATCGCCGCTCCCGATTCGCAGCGCATCGCCTTCTATCGCCTTCTTGACGAGTTCTTCTGAGCGGGACTCTGGGGTTCGAAATGACCGACCAAGCGACGCCCAACCTGCCATCACGAGATTTCGATTCCACCGCCGCCTTCTATGAAAGGTTGGGCTTCGGAATCGTTTTCCGGGACGCCGGCTGGATGATCCTCCAGCGCGGGGATCTCATGCTGGAGTTCTTCGCCCACCCTAGGGGGAGGCTAACTGAAACACGGAAGGAGACAATACCGGAAGGAACCCGCGCTATGACGGCAATAAAAAGACAGAATAAAACGCACGGTGTTGGGTCGTTTGTTCATAAACGCGGGGTTCGGTCCCAGGGCTGGCACTCTGTCGATACCCCACCGAGACCCCATTGGGGCCAATACGCCCGCGTTTCTTCCTTTTCCCCACCCCACCCCCCAAGTTCGGGTGAAGGCCCAGGGCTCGCAGCCAACGTCGGGGCGGCAGGCCCTGCCATAGCCTCAGGTTACTCATATATACTTTAGATTGATTTAAAACTTCATTTTTAATTTAAAAGGATCTAGGTGAAGATCCTTTTTGATAATCTCATGACCAAAATCCCTTAACGTGAGTTTTCGTTCCACTGAGCGTCAGACCCCGTAGAAAAGATCAAAGGATCTTCTTGAGATCCTTTTTTTCTGCGCGTAATCTGCTGCTTGCAAACAAAAAAACCACCGCTACCAGCGGTGGTTTGTTTGCCGGATCAAGAGCTACCAACTCTTTTTCCGAAGGTAACTGGCTTCAGCAGAGCGCAGATACCAAATACTGTCCTTCTAGTGTAGCCGTAGTTAGGCCACCACTTCAAGAACTCTGTAGCACCGCCTACATACCTCGCTCTGCTAATCCTGTTACCAGTGGCTGCTGCCAGTGGCGATAAGTCGTGTCTTACCGGGTTGGACTCAAGACGATAGTTACCGGATAAGGCGCAGCGGTCGGGCTGAACGGGGGGTTCGTGCACACAGCCCAGCTTGGAGCGAACGACCTACACCGAACTGAGATACCTACAGCGTGAGCTATGAGAAAGCGCCACGCTTCCCGAAGGGAGAAAGGCGGACAGGTATCCGGTAAGCGGCAGGGTCGGAACAGGAGAGCGCACGAGGGAGCTTCCAGGGGGAAACGCCTGGTATCTTTATAGTCCTGTCGGGTTTCGCCACCTCTGACTTGAGCGTCGATTTTTGTGATGCTCGTCAGGGGGGCGGAGCCTATGGAAAAACGCCAGCAACGCGGCCTTTTTACGGTTCCTGGCCTTTTGCTGGCCTTTTGCTCACATGTTCTTTCCTGCGTTATCCCCTGATTCTGTGGATAACCGTATTACCGCCATGCAT
